# The association of the ALBI score with disease severity and perinatal outcomes in the preeclampsia spectrum

**DOI:** 10.1186/s12884-026-09049-6

**Published:** 2026-04-13

**Authors:** Cigdem Akcabay, Miray Onat, Neslihan Aslan, Ebru Celik Kavak, Cengiz Sanli, Ibrahim Batmaz, Salih Burcin Kavak

**Affiliations:** 1 Department of Obstetrics and Gynecology, Firat University Faculty of Medicine, Elazig, Turkey; 2 Fethi Sekin City Hospital, Gynecology and Obstetrics Clinic, Elazig, Turkey; 3 Gynecology and Obstetrics Specialist, Elazig, Turkey; 4 Department of Obstetrics and Gynecology, Faculty of Medicine, Mardin Artuklu University, Mardin, Turkey

**Keywords:** Preeclampsia, ALBI score, Disease severity, Gestational age, Perinatal outcomes

## Abstract

**Background:**

This study aimed to investigate the association between the albumin–bilirubin (ALBI) score and disease severity across the preeclampsia spectrum and to evaluate its relationship with perinatal outcomes, with particular emphasis on differences according to gestational age.

**Methods:**

In this retrospective cross-sectional study, medical records of 370 pregnant women diagnosed with preeclampsia between June 2021 and June 2025 were analyzed. Patients were stratified by gestational age at delivery (< 34 weeks and ≥ 34 weeks) and by disease severity (mild-to-moderate preeclampsia vs. severe preeclampsia). Maternal biochemical, hematological, and inflammatory parameters were recorded, and the ALBI score was calculated using serum albumin and total bilirubin levels. Perinatal outcomes included birth weight, Apgar scores, umbilical cord blood gas parameters, admission to the Neonatal Intensive Care Unit (NICU), and length of NICU stay. Receiver operating characteristic analyses were performed to assess the discriminative performance of the ALBI score.

**Results:**

The ALBI score was significantly higher in severe preeclampsia regardless of gestational age and showed superior discriminative performance compared with inflammatory indices, particularly in early-onset disease (< 34 weeks; AUC = 0.702). Higher ALBI scores were associated with adverse perinatal outcomes, including lower birth weight and increased NICU admission. In contrast, inflammatory indices (NLR, PLR, LMR, and SII) did not demonstrate clinically meaningful discrimination of disease severity or neonatal risk. Gestational age at delivery was the primary determinant of NICU length of stay.

**Conclusions:**

The ALBI score is a robust, low-cost, and easily applicable biomarker that reflects biochemical disease severity and perinatal risk across the preeclampsia spectrum, particularly in early-onset severe preeclampsia. Prospective multicenter studies are required to confirm its clinical utility and define its role in future risk stratification algorithms.

## Background

Preeclampsia is a pregnancy-specific hypertensive disorder affecting approximately 2–8% of pregnancies and remains one of the leading causes of maternal and perinatal morbidity and mortality worldwide [1]. It encompasses a broad clinical spectrum, ranging from cases characterized by mild hypertension and proteinuria to severe preeclampsia marked by multiorgan dysfunction, reflecting the heterogeneity of the disease [2].

The pathophysiology of preeclampsia is described as a multistage process that begins with abnormal placentation and impaired uteroplacental perfusion and subsequently progresses to a systemic maternal response dominated by endothelial dysfunction, oxidative stress, and widespread inflammation [3]. This systemic response results in functional and biochemical alterations across multiple organ systems, particularly the liver, and may be accompanied by reduced serum albumin levels and disturbances in bilirubin metabolism, which contribute to the clinical manifestations of the disease. Various inflammatory indices derived from routine hematological parameters, such as the neutrophil-to-lymphocyte ratio (NLR), platelet-to-lymphocyte ratio (PLR), lymphocyte-to-monocyte ratio (LMR), and systemic immune-inflammation index (SII), have been investigated as potential markers of systemic inflammation in various clinical conditions, including hypertensive disorders of pregnancy [4, 5]. These indices are thought to reflect the balance between pro-inflammatory and anti-inflammatory immune responses; however, findings regarding their clinical relevance in preeclampsia remain inconsistent. The albumin–bilirubin (ALBI) score is an evidence-based scoring system originally developed to provide an objective assessment of liver function in patients with hepatocellular carcinoma, based on serum albumin and total bilirubin levels [6]. Currently, the ALBI score is widely used not only to evaluate hepatic reserve but also as a standard measure for detecting early and subclinical alterations in liver function [7]. Its ability to reflect even subtle biochemical fluctuations renders the ALBI score a potentially valuable biomarker in clinical conditions characterized by systemic stress and accompanying organ dysfunction.

Given that preeclampsia is a systemic disorder characterized by multiorgan involvement, the ALBI score is hypothesized to have the potential to reflect disease severity and perinatal risk across this clinical spectrum. However, studies evaluating the ALBI score according to preeclampsia subtypes and gestational age groups remain limited in the current literature.

The aim of this study was to assess the association between the ALBI score and disease severity and perinatal outcomes within the preeclampsia spectrum and to examine whether this relationship varies according to gestational age. The findings of this study are expected to provide insight into the potential use of the ALBI score as a complementary biochemical marker that may contribute to clinical risk stratification in preeclampsia.

## Materials and methods

This retrospective cross-sectional study was conducted at the Department of Obstetrics and Gynecology, Fırat University Faculty of Medicine, after obtaining approval from the local Institutional Ethics Committee (Approval No. 2025/13–26), and in accordance with the principles of the Declaration of Helsinki. Due to the retrospective design of the study and the use of anonymized patient data, the requirement for written informed consent was waived by the ethics committee. The medical records of pregnant women who were admitted to our clinic with a diagnosis of preeclampsia (PE) between June 2021 and June 2025 were retrospectively reviewed. During the study period, a total of 480 cases within the preeclampsia spectrum were identified. Of these, 370 cases with complete and adequate medical records were included in the final analysis.

A total of 480 cases diagnosed with preeclampsia were initially identified, of which 110 were excluded due to incomplete laboratory data or missing delivery outcome records. A review of the available obstetric and demographic data indicated that there were no meaningful differences between the excluded cases and the study cohort with respect to maternal age, gravidity, or parity. Patients were stratified into four subgroups according to gestational age at delivery (< 34 weeks and ≥ 34 weeks) and disease severity: <34 weeks mild-to-moderate preeclampsia, < 34 weeks severe preeclampsia (sPE), ≥ 34 weeks mild-to-moderate preeclampsia, and ≥ 34 weeks severe preeclampsia (sPE).

Review of the medical records confirmed that all cases included in the study met the diagnostic criteria for preeclampsia. The diagnoses of mild-to-moderate preeclampsia and severe preeclampsia were established in accordance with the criteria defined by the American College of Obstetricians and Gynecologists [2]. Accordingly, in patients diagnosed with mild-to-moderate preeclampsia, blood pressure measurements obtained at least four hours apart after 20 weeks of gestation documented systolic blood pressure ≥ 140 mmHg and/or diastolic blood pressure ≥ 90 mmHg in previously normotensive women. In cases diagnosed with severe preeclampsia, systolic blood pressure ≥ 160 mmHg and/or diastolic blood pressure ≥ 110 mmHg was recorded.

Findings accompanying hypertension included proteinuria of ≥ 300 mg in a 24-hour urine collection, a protein-to-creatinine ratio ≥ 0.3, or, when required, ≥ 2 + proteinuria on dipstick testing. In the absence of proteinuria, the presence of at least one sign of new-onset maternal organ dysfunction in association with hypertension was documented, including thrombocytopenia (platelet count < 100,000/mm³), elevated serum creatinine (> 1.1 mg/dL or a doubling of baseline values), liver transaminase levels more than twice the upper limit of normal, pulmonary edema, or new-onset headache or visual disturbances not attributable to alternative diagnoses.

The fulfillment of the same ACOG criteria was also confirmed in the medical records for the identification of the severe preeclampsia (sPE) group. Accordingly, patients in the sPE group had documented systolic blood pressure ≥ 160 mmHg or diastolic blood pressure ≥ 110 mmHg, along with clinical evidence of thrombocytopenia (platelet count < 100,000/mm³), significant hepatic dysfunction, elevated serum creatinine levels, pulmonary edema, or new-onset headache or visual disturbances refractory to medical treatment.

Patients were excluded from the study if they had chronic or gestational hypertension, a history of chronic inflammatory or systemic diseases (e.g., systemic lupus erythematosus or rheumatoid arthritis), evidence of acute infection (such as tonsillitis or urinary tract infection), pregestational or gestational diabetes mellitus, multiple pregnancy, or a history of smoking. Cases with incomplete laboratory data or missing delivery outcome records were also excluded from the analysis. To minimize potential confounding factors, patients with chronic hypertension or superimposed preeclampsia on chronic hypertension were excluded from the study. Although some of the evaluated cases exhibited clinical and laboratory findings of hepatic dysfunction that may fall within the spectrum of severe preeclampsia, patients meeting the classical diagnostic criteria for HELLP syndrome were not analyzed as a separate subgroup. This was primarily because the study was designed to evaluate the biochemical differences between the mild-to-moderate preeclampsia and severe preeclampsia groups.

Laboratory parameters obtained at the time of admission for each patient—including complete blood count, white blood cell subtypes, platelet count, serum creatinine, urea, aspartate aminotransferase, alanine aminotransferase, albumin, and total bilirubin levels—were extracted from the medical records. Using these data, inflammatory indices were calculated, including the neutrophil-to-lymphocyte ratio (NLR), platelet-to-lymphocyte ratio (PLR), lymphocyte-to-monocyte ratio (LMR), systemic immune-inflammation index (SII; platelet × neutrophil/lymphocyte), and aggregate index of systemic inflammation (AISI; neutrophil × monocyte × platelet/lymphocyte). In addition, the albumin–bilirubin (ALBI) score was calculated based on serum albumin and total bilirubin levels.

The ALBI score was calculated using the following formula [4]:

$$\mathrm{ALBI}=\left(\log_{10}\;\mathrm{bilirubin}\;\left[\mathrm{\mu}{mol}/\mathrm L\right]\times0.66\right)+\left(\mathrm{albumin}\;\left[\mathrm g/\mathrm L\right]\times-0.085\right).$$

Neonatal outcomes were obtained from postnatal clinical records and included birth weight, Apgar scores recorded at 1 and 5 min after delivery, and umbilical cord blood gas parameters measured immediately after birth (pH, base excess, and lactate). Data regarding NICU admission status and length of stay, as well as admission details and duration of intensive care for neonates requiring NICU care, were retrieved from the hospital’s neonatal intensive care unit records.

The primary outcome measures were defined as differences in maternal biochemical and hematological parameters (platelet count, albumin, total bilirubin, and ALBI score) and perinatal outcomes (NICU admission, length of NICU stay, and neonatal clinical parameters) according to the presence of severe preeclampsia in pregnancies stratified as < 34 weeks and ≥ 34 weeks of gestation.

### Statistical analysis

Descriptive statistics were presented as mean ± standard deviation (SD), median (minimum–maximum), frequency, and percentage, as appropriate. The distribution of continuous variables was assessed using the Kolmogorov–Smirnov and Shapiro–Wilk tests. Normally distributed independent continuous variables were compared using the independent samples t-test, whereas non-normally distributed variables were analyzed using the Mann–Whitney U test. Categorical variables were compared using the chi-square test, and Fisher’s exact chi-square test was applied when expected cell counts were insufficient.

Receiver operating characteristic (ROC) curve analysis was performed to evaluate the discriminative performance of the ALBI score for severe preeclampsia in pregnancies stratified as < 34 and ≥ 34 weeks of gestation. The area under the curve (AUC) and 95% confidence intervals were calculated. Optimal cut-off values were determined, and sensitivity, specificity, positive predictive value (PPV), and negative predictive value (NPV) were reported. All statistical analyses were conducted using SPSS software version 28.0 (IBM Corp., Armonk, NY, USA), and a *p* value < 0.05 was considered statistically significant.

### Power analysis

The primary endpoint of the study was defined as the difference in ALBI scores between the mild-to-moderate preeclampsia (PE) and severe preeclampsia (sPE) groups at < 34 weeks of gestation. In this subgroup, mean ALBI scores were − 2.6 ± 0.4 (*n* = 103) in the PE group and − 2.3 ± 0.4 (*n* = 100) in the sPE group; based on these values, Cohen’s effect size was calculated as d = 0.75. Using G*Power version 3.1 (Heinrich Heine University Düsseldorf, Germany), a post hoc power analysis was performed for a two-tailed independent samples t-test with α = 0.05 and d = 0.75, yielding a statistical power exceeding 99% for the < 34-week subgroup.

When the same analysis was repeated for the ≥ 34-week subgroup using an effect size of d = 0.47 with sample sizes of n₁ = 112 and n₂ = 55, the estimated statistical power was approximately 80%. These findings indicate that the available sample size was sufficient to detect clinically meaningful differences in ALBI scores.

## Results

In this study, cases of preeclampsia were stratified into two main groups according to gestational age at delivery: ≥34 weeks and < 34 weeks. Each gestational age group was further subdivided into mild-to-moderate preeclampsia and severe preeclampsia, yielding a total of four clinical groups. The results are presented according to this classification, and comparisons of hematological, biochemical, and inflammatory parameters, as well as neonatal outcomes, are summarized in the corresponding tables.

With respect to obstetric and demographic characteristics, no significant differences were observed among the groups in terms of maternal age, gravidity, parity, or number of living children. However, in the ≥ 34-week severe preeclampsia group, gestational age at the time of diagnosis was significantly lower compared with the corresponding mild-to-moderate preeclampsia group (*p* = 0.013). This finding suggests that severe preeclampsia leads to earlier clinical presentation even in late gestation. In contrast, in the < 34-week group, none of the evaluated parameters differed significantly according to the type of preeclampsia (*p* > 0.05). These data are presented in Table 1.

**Table 1:** Demographic and obstetric characteristics of the mild-to-moderate preeclampsia and severe preeclampsia groups

Parameters	≥34 Mild-to-moderate PE (*n* = 112) Mean ± SD	≥34 Severe PE (*n* = 55) Mean ± SD	*p* value*		<34 Mild-to-moderate PE (*n* = 103) Mean ± SD	<34 Severe PE (*n* = 100) Mean ± SD	*p* value*
Gestational age at diagnosis (weeks)	36.7 ± 1.5	36.1 ± 1.5		**0.013**	30.1 ± 2.8	29.5 ± 3.1	0.198
Age (years)	30.3 ± 6.7	30.3 ± 6.8		0.904	31.7 ± 6.8	30.9 ± 6.0	0.325
Gravidity (number)	2.54 ± 1.77	2.59 ± 1.93		0.859	2.58 ± 1.85	2.63 ± 1.83	0.768
Parity (number)	1.10 ± 1.38	1.24 ± 1.50		0.546	1.22 ± 1.40	1.26 ± 1.45	0.920
Number of living children (number)	1.91 ± 1.22	2.03 ± 1.48		0.712	1.74 ± 1.15	1.95 ± 1.38	0.595

*Between-group comparisons were performed using the Mann–Whitney U test. *n* number, *PE* preeclampsia, *Mean* ± *SD* mean ± standard deviation, * *p* < 0,05

Bold values indicate statistically significant differences (p < 0.05)

When biochemical and inflammatory parameters were evaluated, platelet count, albumin, bilirubin, and the ALBI score differed significantly between mild-to-moderate preeclampsia and severe preeclampsia in both gestational age strata (< 34 weeks and ≥ 34 weeks). In the severe preeclampsia groups, serum albumin levels were lower, whereas bilirubin levels and ALBI scores were higher (all *p* < 0.05). The ALBI score demonstrated a markedly stronger discriminative capacity than albumin and platelet levels alone in reflecting the biochemical severity of severe preeclampsia and in predicting neonatal outcomes.

In contrast, inflammatory markers (NLR, PLR, LMR, and SII) did not exhibit significant discriminatory ability between the groups, with only a minimal change in PLR observed in the ≥ 34-week group. These findings are presented in Table 2.

**Table 2:** Hematological, biochemical, and inflammatory parameters of the mild-to-moderate preeclampsia and severe preeclampsia groups

Parameters	≥ 34 Mild-to-moderate PE (*n* = 112) Mean ± SD	≥ 34 Severe PE (*n* = 55) Mean ± SD	*p* value*	< 34 Mild-to-moderate PE (*n* = 103) Mean ± SD	< 34 Severe PE (*n* = 100) Mean ± SD	*p* value*
Neutrophil (10³/µL)	8.0 ± 2.4	8.4 ± 3.4	0.927	8.7 ± 3.2	9.3 ± 3.7	0.406
Lymphocyte (10³/µL)	2.1 ± 0.7	2.1 ± 0.8	0.631	2.1 ± 0.8	2.0 ± 0.9	0.064
Monocyte (10³/µL)	0.61 ± 0.22	0.61 ± 0.24	0.777	0.65 ± 0.30	0.61 ± 0.32	0.390
Platelet (10³/µL)	262.8 ± 77.9	220.4 ± 71.5	**0.005**	248.4 ± 69.6	215.2 ± 97.4	**0.004**
Albumin (g/L)	36.2 ± 3.6	33.6 ± 7.4	**0.013**	36.5 ± 3.8	34.6 ± 3.6	**< 0.001**
Total Bilirubin (µmol/L)	8.4 ± 4.4	8.6 ± 8.0	0.323	6.2 ± 2.3	10.7 ± 10.2	**< 0.001**
ALBI	–2.5 ± 0.3	–2.3 ± 0.6	**0.039**	–2.6 ± 0.4	–2.3 ± 0.4	**< 0.001**
NLR	4.3 ± 2.8	4.8 ± 3.3	0.791	4.9 ± 3.5	6.4 ± 5.3	0.098
PLR	134.9 ± 56.9	117.4 ± 53.0	**0.044**	134.3 ± 71.4	128.3 ± 71.0	0.582
LMR	3.9 ± 1.6	3.8 ± 1.7	0.767	4.0 ± 2.5	4.0 ± 2.3	0.989
SII	1122 ± 775	1057 ± 781	0.187	1241 ± 1014	1242 ± 957	0.870

*Between-group comparisons were performed using the Mann–Whitney U test. *n* number, *Mean* ± *SD* mean ± standard deviation, *PE* preeclampsia, *ALBI* albumin–bilirubin score, *NLR* neutrophil-to-lymphocyte ratio, *PLR* platelet-to-lymphocyte ratio, *LMR* lymphocyte-to-monocyte ratio, *SII* systemic immune-inflammation index, * *p* < 0,05

Bold values indicate statistically significant differences (p < 0.05)

When the discriminative performance of the ALBI score for severe preeclampsia was evaluated, it demonstrated a moderate ability to distinguish severe preeclampsia in pregnancies at ≥ 34 weeks of gestation (AUC = 0.599, *p* = 0.039) and a good discriminatory performance in pregnancies at < 34 weeks of gestation (AUC = 0.702, *p* < 0.001). These results are presented in Table 3.

**Table 3:** Diagnostic performance of the ALBI score in distinguishing severe preeclampsia according to gestational age

Parameters	≥34 weeks			< 34 weeks		
	AUC	%95 CI	*p* value	AUC	%95 CI	*p* value
ALBI (raw value)	0.599	0.505–0.692	0.039	0.702	0.630–0.773	< 0.001
Without applying a cut-off	–	–	–	–	–	–
	**Cut-off**	**Sensitivity**	**Specificity**	**Cut-off**	**Sensitivity**	**Specificity**
ALBI (cut-off − 2.60)	–2.60	70.9%	44.6%	− 2.60	77.0%	52.4%
	**PPV**	**NPV**		**PPV**	**NPV**	
predictive values for a cut-off of − 2.60	38.6%	75.8%		61.1%	70.1%	

*ALBI* albumin–bilirubin score, *AUC* area under the curve, *CI* confidence interval, *PPV* positive predictive value, *NPV* negative predictive value. A cut-off value of − 2.60 was used

Bold values indicate statistically significant differences (p < 0.05)

A cut-off value of − 2.60 provided higher sensitivity and positive predictive value in the < 34-week group, suggesting that hepatocellular stress is more pronounced in the early stages of severe preeclampsia. This finding supports the potential clinical utility of the ALBI score as a biomarker for distinguishing early-onset severe preeclampsia, particularly before 34 weeks of gestation.

When perinatal outcomes were evaluated, the severe preeclampsia group at ≥ 34 weeks of gestation had significantly lower birth weight (*p* = 0.003) and a higher rate of NICU admission (*p* = 0.001) compared with the mild-to-moderate preeclampsia group; however, no significant differences were observed between the two groups with respect to Apgar scores, umbilical cord blood pH, lactate levels, or base excess. These findings are presented in Table 4.

**Table 4:** Comparison of neonatal outcomes between the mild-to-moderate preeclampsia and severe preeclampsia groups according to gestational age

Parameters	≥34 Mild-to-moderate PE (*n* = 112) Mean ± SD	≥34 Severe PE (*n* = 55) Mean ± SD	*p* value	<34 Mild-to moderate PE (*n* = 103) Mean ± SD	<34 Severe PE (*n* = 100) Mean ± SD	*p* value
Neonatal BW (grams)	2663 ± 641	2324 ± 561	**0.003** ^**m**^	1247 ± 593	1191 ± 529	0.645^m^
Birth weight cen. (%)	41.3 ± 25.7	27.4 ± 23.6	**0.006** ^**m**^	22.2 ± 31.9	20.3±29.1	0.472^m^
1-minute Apgar score median (IQR)	7 (5–8)	6 (5–7)	0.264^m^	4 (3–6)	4 (3–5)	0.549 ^m^
5-minute Apgar score median (IQR)	9 (8–9)	8 (7–9)	0.236^m^	6 (5–8)	6 (5–8)	0.649 ^m^
Umbl. cord blood pH	7.3 ± 0.1	7.3 ± 0.1	0.369 ^m^	7.3 ± 0.1	7.3 ± 0.1	0.082 ^m^
Base deficit	–2.2 ± 3.1	− 3.0 ± 3.1	0.117 ^m^	− 3.0 ± 3.9	− 3.8 ± 4.1	0.078 ^m^
Lactate (mmol/L)	2.8 ± 1.9	3.2 ± 2.3	0.287 ^m^	4.2 ± 3.9	4.7 ± 4.3	0.196 ^m^
NICU admission (Yes)	29.5%	57.7%	**0.001** ^**X²**^	98.0%	97.9%	1.000 ^X²^
Length of NICU stay (days)	9.5 ± 10.0	11.8 ± 7.6	0.092 ^m^	27.7 ± 23.5	31.7 ± 23.6	0.260 ^m^

Continuous variables are expressed as mean ± standard deviation (SD), except for Apgar scores, which are presented as median (interquartile range, IQR). Continuous variables were compared using the Mann–Whitney U test (m), and categorical variables were analyzed using the chi-square test (χ²). *n* number, Mean ± SD: mean ± standard deviation. *PE* preeclampsia, *BW* birth weight, *cen* centile, *Umbl* Umbilical, *NICU* Neonatal Intensive Care Unit, Birth weight centiles were calculated according to gestational age

Bold values indicate statistically significant differences (p < 0.05)

In the < 34-week group, neonatal outcomes in the severe preeclampsia group were not significantly worse, and NICU admission rates exceeded 98% across all subgroups, largely reflecting the effect of prematurity.

Regardless of gestational age, NICU length of stay was numerically longer in cases of severe preeclampsia; however, this difference did not reach statistical significance, suggesting that the duration of NICU hospitalization was primarily determined by prematurity rather than disease severity.

When the effects of biochemical, hematological, and inflammatory parameters on NICU admission and length of NICU stay were evaluated, the ALBI score emerged as the strongest predictor of NICU admission among all assessed biomarkers. Consistent with this finding, serum albumin and total bilirubin levels, when evaluated individually, also demonstrated significant associations with NICU admission in both gestational age groups (all *p* < 0.05). In contrast, none of the inflammatory markers (NLR, PLR, LMR, and SII) showed a significant effect on either NICU admission or duration of NICU stay.

Gestational age at delivery was identified as the most important determinant of NICU length of stay, with the impact of prematurity outweighing that of all evaluated biomarkers. The effects of the assessed parameters on NICU admission and NICU length of stay are presented in Table 5.

**Table 5:** Association of biochemical, hematological, and inflammatory parameters with Neonatal Intensive Care Unit (NICU) admission and length of NICU stay

Biomarkers	Parameters	NICU admission — Association with	*p*-value*	Association with length of NICU stay	*p*-value*	Clinical interpretation
Biochemical	Albumin	Significant	**0.013/<0.001**	Weak association	>0.05	Strong predictor of NICU admission; no effect on length of stay.
	Bilirubin	Significant in <34 weeks	0.323/**<0.001**	NS	>0.05	Associated with severe preeclampsia; strong predictor of NICU admission; no effect on length of stay.
ALBI	Composite	*Strong association*	**0.039/<0.001**	*Weak association*	> 0.05	Strongest biochemical predictor of NICU admission; no effect on length of stay.
Hematological	Platelet	*Significant*	**0.005/0.004**	NS	> 0.05	Associated with severe preeclampsia; weak predictor of NICU admission; no effect on length of stay.
Inflammatory**	NLR	NS	> 0.05	NS	> 0.05	Not predictive
	PLR	*Minimal difference*	**0.044**	NS	> 0.05	Limited clinical value
	LMR	NS	> 0.05	NS	> 0.05	Not predictive
	SII	NS	> 0.05	NS	> 0.05	Not predictive
Neonatal	Gasetational age	*Strongest determinant*	**< 0.001**	*Strongest determinant*	**< 0.001**	Regardless of biomarkers, the presence of prematurity was the main determinant.

*p* value*. Based on the Mann–Whitney U test derived from comparisons between the ≥ 34 and < 34 weeks groups presented in Tables 2, 3 and 4. *ALBI* albumin–bilirubin score, *NS* not significant, *NLR* neutrophil-to-lymphocyte ratio, *PLR* platelet-to-lymphocyte ratio, *LMR* lymphocyte-to-monocyte ratio, *SII* systemic immune-inflammation index, *NICU* Neonatal Intensive Care Unit

** None of the inflammatory parameters (NLR, PLR, LMR, SII) showed a significant association with NICU admission or length of NICU stay

Bold values indicate statistically significant differences (p < 0.05)

## Discussion

This study demonstrates that the ALBI score is a meaningful biomarker for assessing biochemical involvement across the preeclampsia spectrum and may be associated with perinatal risks. The elevation of the ALBI score in severe preeclampsia reflects hepatocellular stress and endothelial dysfunction in an integrated manner. Its higher discriminative performance particularly in pregnancies < 34 weeks of gestation (AUC = 0.702) suggests that hepatic involvement may be more pronounced in early-onset disease. In contrast, the lack of significant discriminatory performance of inflammatory indices indicates that the ALBI score may represent a relatively stronger biochemical indicator for predicting both disease severity and perinatal risks, such as low birth weight and increased NICU requirement.

Although the pathophysiology of preeclampsia has not been fully elucidated, it is considered a multifactorial process [8]. Preeclampsia is widely accepted as a two-stage disorder, beginning with abnormal placentation in early pregnancy and subsequently evolving into a “maternal syndrome” in later gestation, driven by increased circulating antiangiogenic factors in the maternal circulation [9]. Placental ischemia and the resulting elevation in soluble fms-like tyrosine kinase-1 (sFlt-1) and soluble endoglin (sEng) levels trigger endothelial dysfunction, oxidative stress, and systemic inflammation, ultimately leading to multiorgan involvement [10].

The inflammatory response induced by preeclampsia activates cyclooxygenase (COX) pathways, resulting in increased thromboxane A₂ (TxA₂) production and reduced endothelial prostacyclin (PGI₂) synthesis [11]. Consequently, this inflammatory cascade contributes substantially to the clinical phenotype of preeclampsia.

Although several studies have been published in recent years examining the association between inflammatory indices and preeclampsia, the clinical relevance of the findings has remained limited. In a study published by Conde-Rico et al. in 2023, among the evaluated inflammatory parameters, only the lymphocyte-to-monocyte ratio (LMR) showed a weak association with the presence of preeclampsia; however, none of the inflammatory indices demonstrated significant predictive value for severe preeclampsia [12]. In that study, the discriminative performance of LMR was low (AUC 0.56–0.57), indicating limited clinical applicability. These findings suggest that inflammatory indices have limited value in determining the severity of preeclampsia.

Similarly, in a large-scale cohort analysis conducted by Yao et al., absolute immune cell counts (neutrophils, lymphocytes, and platelets) measured between 11 and 28 weeks of gestation were reported to be associated with an increased risk of preeclampsia, whereas derived inflammatory indices (NLR, PLR, LMR, and SII) showed no significant association with preeclampsia [13].

Moreover, the absence of a causal contribution of immune cell counts to preeclampsia in the Mendelian randomization analyses further supports the limited capacity of these parameters to reflect disease severity. Consistent with these findings, none of the inflammatory indices demonstrated significant differences among preeclampsia subgroups in our cohort, fully aligning with the existing literature and underscoring the low clinical utility of inflammatory indices in determining the biological severity of preeclampsia.

The ALBI score is an objective and reliable parameter reflecting liver function, based on serum albumin and bilirubin levels. It has been shown to be a more accurate and robust predictor than classical scoring systems such as Child–Pugh and MELD, both for assessing hepatic reserve and for predicting the risk of post-hepatectomy liver failure and mortality [14, 15].

Beyond hepatology, the ALBI score—which integrates hepatic reserve with systemic physiological stress—has demonstrated strong predictive accuracy across diverse clinical settings. For instance, in a study by Inoue-Yuri et al. published in 2022, optimization of cut-off values for the ALBI-PLT score significantly improved the safe exclusion of high-risk esophageal varices, underscoring the robustness of the ALBI score’s biological sensitivity [16].

Another study supporting the hypothesis that the ALBI score may serve as a strong risk marker in conditions characterized by systemic organ involvement, such as preeclampsia, is the large cohort analysis published by Wang et al. in 2024. In that study, the ALBI score demonstrated a linear and independent association with all-cause mortality among intensive care unit patients with heart failure; increasing ALBI levels were associated with significantly higher 30-day and 365-day mortality risks, and the ALBI score remained an independent risk marker even after multivariable adjustment [17].

These findings indicate that the ALBI score is a robust biomarker reflecting both hepatocellular function and systemic physiological stress, making it potentially useful in conditions involving multiorgan dysfunction such as preeclampsia. Consistent with this concept, in our study the ALBI score demonstrated clear superiority over inflammatory indices in discriminating severe preeclampsia in both the < 34-week and ≥ 34-week subgroups and emerged as the only biochemical marker with a significantly higher discriminative performance, particularly at earlier gestational ages.

Publications evaluating the relationship between the ALBI score and pregnancy remain limited. In our PubMed database search, only five studies were identified in which the ALBI score was applied in pregnancy-related conditions. Among these, a study published by Soysal et al. in 2025 reported that hepatocellular stress becomes pronounced in hyperemesis gravidarum (HG), as reflected by significant increases in both the ALBI and APRI scores. In that study, the ALBI score demonstrated a statistically significant, albeit limited, diagnostic performance in distinguishing HG from a control group [18]. These findings suggest that the ALBI score may serve as a sensitive biochemical marker capable of reflecting hepatic functional impairment in acute obstetric conditions.

In a study including 165 pregnancies in women with chronic liver disease, Gonsalkorala et al. reported that the ALBI score outperformed conventional liver function scores such as MELD and APRI in predicting pregnancy outcomes. Notably, the preconception ALBI score demonstrated the highest accuracy for predicting live birth, with an AUC of 0.741 [19].

These findings suggest that the ALBI score may have prognostic value in pregnancy as an integrative biomarker reflecting not only hepatic reserve but also systemic physiological stress. Consistent with this concept, in our preeclampsia cohort the ALBI score demonstrated clear superiority in discriminating severe preeclampsia in both the < 34-week and ≥ 34-week subgroups and was significantly elevated in severe preeclampsia in parallel with decreased albumin and increased bilirubin levels. Moreover, the fact that ALBI was the only biochemical marker to exhibit strong discriminatory performance at earlier gestational ages (AUC = 0.702) supports the notion that this score directly reflects hepatocellular stress and endothelial dysfunction within the pathophysiology of preeclampsia. Taken together, these studies indicate that the ALBI score is not limited to chronic liver disease–specific contexts in pregnancy, but rather emerges as a valuable biomarker capable of sensitively capturing maternal hepatic involvement across diverse pathophysiological conditions and of predicting perinatal risk. Previous studies have reported that neonates born to mothers with preeclampsia may develop transient hematological abnormalities, including neutropenia and thrombocytopenia, particularly in cases of severe disease. These findings further support the concept that severe preeclampsia is associated not only with maternal multiorgan involvement but also with systemic effects on the fetus and the neonatal hematological profile [20, 21].

In addition, studies focusing on intrahepatic cholestasis of pregnancy (ICP) further support the ability of the ALBI score to sensitively reflect liver-related biochemical stress during pregnancy. Obut et al. reported that ALBI, MELD, and APRI scores were significantly elevated in cases of ICP both in the first trimester and at the time of diagnosis; among these scores, the ALBI score demonstrated the highest predictive accuracy, with a first-trimester AUC of approximately 0.83, and emerged as a strong predictor of ICP development [22]. These findings indicate that the capacity of the ALBI score to detect early biochemical deterioration in obstetric settings is not confined to liver-specific diseases, but rather extends to a broader range of pregnancy-related pathologies, underscoring its potential value as a versatile biomarker in pregnancy.

This study is among the limited number of investigations to separately evaluate the performance of the ALBI score in assessing biochemical disease severity across the preeclampsia spectrum in both early- and late-onset subgroups. The large sample size, the use of a homogeneous dataset derived from standardized laboratory measurements within a single center, and the clear distinction between mild-to-moderate preeclampsia and severe preeclampsia subgroups based on well-defined diagnostic criteria enhance the methodological strength of the study. The direct comparison of the ALBI score with inflammatory indices and the concurrent evaluation of its association with perinatal outcomes provide a comprehensive perspective on the clinical applicability of the findings. Moreover, the study yields novel evidence suggesting that the ALBI score may reflect not only maternal biochemical involvement but also fetal risk.

The present study has several limitations that should be acknowledged. First, the retrospective design limits the ability to establish causal relationships and does not allow complete standardization of the timing of laboratory measurements. In addition, as the study was conducted in a single center, the generalizability of the findings to broader populations may be limited. Furthermore, the ALBI score was calculated based on laboratory values obtained at a single time point, which prevented the evaluation of potential longitudinal changes during the course of the disease. Robust evidence from prospective studies is required before the ALBI score can be integrated into routine clinical protocols.

Another limitation of this study is that, due to its retrospective design, bilirubin fractions (direct and indirect bilirubin) were not consistently available for all patients. Therefore, the ALBI score was calculated using only total bilirubin levels. Future prospective studies evaluating bilirubin fractions separately may provide further insight into whether bilirubin elevation reflects hepatocellular dysfunction or alternative mechanisms such as intravascular hemolysis. In addition, standardized 24-hour proteinuria measurements were not consistently available for all patients, which precluded analysis of the association between the ALBI score and the degree of proteinuria.

## Conclusions

This study demonstrates that the albumin–bilirubin (ALBI) score is a robust and sensitive biochemical marker reflecting disease severity across the preeclampsia spectrum. Higher ALBI scores were associated with severe preeclampsia and adverse perinatal outcomes, particularly in early-onset disease, whereas commonly used inflammatory indices showed limited clinical discrimination. These findings suggest that the ALBI score may serve as a practical and low-cost adjunct for biochemical risk stratification in preeclampsia, although larger prospective multicenter studies are needed to confirm its clinical utility.

## Data Availability

Data is provided within the main manuscript file.
